# Thrombus in Transit: A Two-Case Series Highlighting Clinical Challenges and Outcomes

**DOI:** 10.7759/cureus.104036

**Published:** 2026-02-21

**Authors:** Garrett A Perchetti, Tambi Isaac, Samy Matta, Hnin Aye, Roxana Lazarescu

**Affiliations:** 1 Internal Medicine, Wyckoff Heights Medical Center, New York, USA; 2 Medical Academy, Kabardino-Balkarian State University, Nalchik, RUS

**Keywords:** anticoagulation, clot-in-transit, embolectomy, pulmonary embolism, pulmonary embolism treatment, surgical embolectomy, surgical thrombectomy, thrombolysis, thrombus-in-transit, venous thromboembolism

## Abstract

Thrombus in transit (TIT), defined as mobile right atrial or right ventricular thrombi often associated with acute pulmonary embolism (PE), carries a high risk of morbidity and mortality. Optimal management remains controversial, with options including anticoagulation, systemic thrombolysis, surgical embolectomy, and percutaneous thrombectomy. We describe two elderly women with a history of thromboembolic disease who presented with acute decompensation in the setting of TIT and recurrent PE. The first case involved an 81-year-old female with massive bilateral PE and right atrial thrombus who underwent suction thrombectomy but died perioperatively. The second case involved a 74-year-old female with prior right atrial thrombectomy for TIT who presented with acute hypoxemic respiratory failure, large left pleural effusion, recurrent PE, and septic shock; despite anticoagulation with Argatroban and intensive care support, she developed cardiac arrest and died. These cases underscore the diagnostic and therapeutic challenges of TIT, particularly in elderly patients with multiple comorbidities. Early recognition, multidisciplinary coordination, and individualized management remain essential, but outcomes can be poor despite aggressive interventions.

## Introduction

Thrombus in transit (TIT) refers to a mobile thrombus visualized in the right atrium/ventricle, mainly in the absence of atrial fibrillation, or in the superior/inferior vena cava, usually migrating from the systemic venous system toward the pulmonary circulation. It represents a rare but life-threatening phenomenon, seen in approximately 4-18% of patients with acute pulmonary embolism (PE). Mortality rates are significantly higher than in isolated PE, estimated at about 40%, especially when associated with right ventricular dysfunction or hemodynamic instability [1,2].

Despite recognition of its prognostic significance, the optimal management of TIT remains uncertain. Treatment options include anticoagulation alone, systemic thrombolysis, surgical thrombectomy, and percutaneous mechanical thrombectomy. Randomized trials are lacking, and therapeutic decisions are typically based on institutional expertise, patient comorbidities, and hemodynamic status [3].

We present two cases of elderly women with recurrent venous thromboembolism and TIT, both of whom suffered poor outcomes despite escalation of care, and we discuss these cases in the context of the current literature.

## Case presentation

Case 1

An 81-year-old woman with a history of hypertension and dementia presented with an acute onset of dyspnea, tachycardia, and hypoxemia. Her symptoms began abruptly while ambulating to the bathroom, representing a marked decline from her baseline functional status. She was largely homebound and ambulated short distances with a cane or walker. According to her daughter, she had no personal or family history of venous thromboembolism or malignancy, and she had not undergone recent surgery or hospitalization.

**Figure 1:**
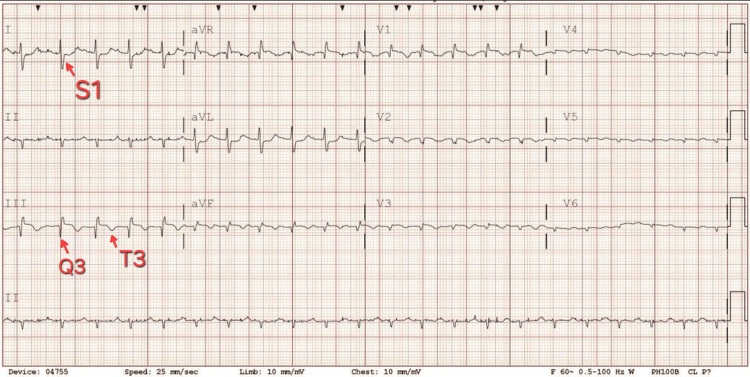
Sinus tachycardia and S1, Q3, T3 sign for pulmonary embolism, low voltage, and poor R wave progression. S_1_: S wave in lead I; Q_3_: Q wave in lead III; T_3_: T-wave inversion in lead III

**Figure 2:**
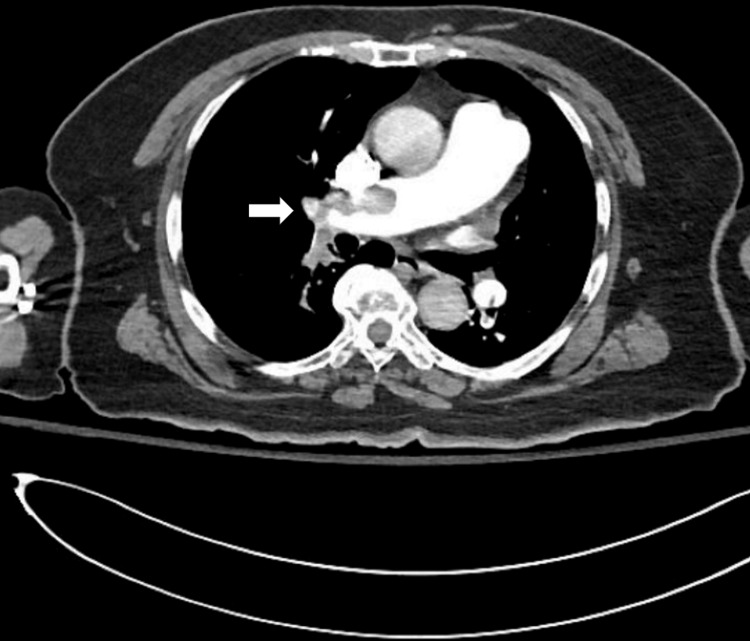
CTPA is showing a thrombus in the right pulmonary artery (white arrow). CTPA: computed tomography pulmonary angiogram

On arrival to the emergency department, she was tachycardic to 140 beats per minute and hypotensive and required supplemental oxygen. Laboratory findings were significant for lactic acidosis and elevated B-type natriuretic peptide (BNP) and troponin, as shown in Table 1. EKG was remarkable for sinus tachycardia, and S wave in lead I (S_1_), a Q wave in lead III (Q_3_), and T-wave inversion in lead III (T_3_) sign for PE, low voltage (chest limb criteria), and poor R wave progression, as shown in Figure 1. CT pulmonary angiography was significant for thrombus in the right pulmonary artery (Figure 2).

**Table 1:** Laboratory results at admission. BNP: B-type natriuretic peptide

Lab test	Result	Reference range
Lactate	7.2 mmol/L	0.5-2.2 mmol/L
BNP	203 pg/mL	<100 pg/mL
High-sensitivity troponin	93 ng/L	<14 ng/L

Transthoracic Echocardiogram (TTE) and Doppler Studies

**Figure 3:**
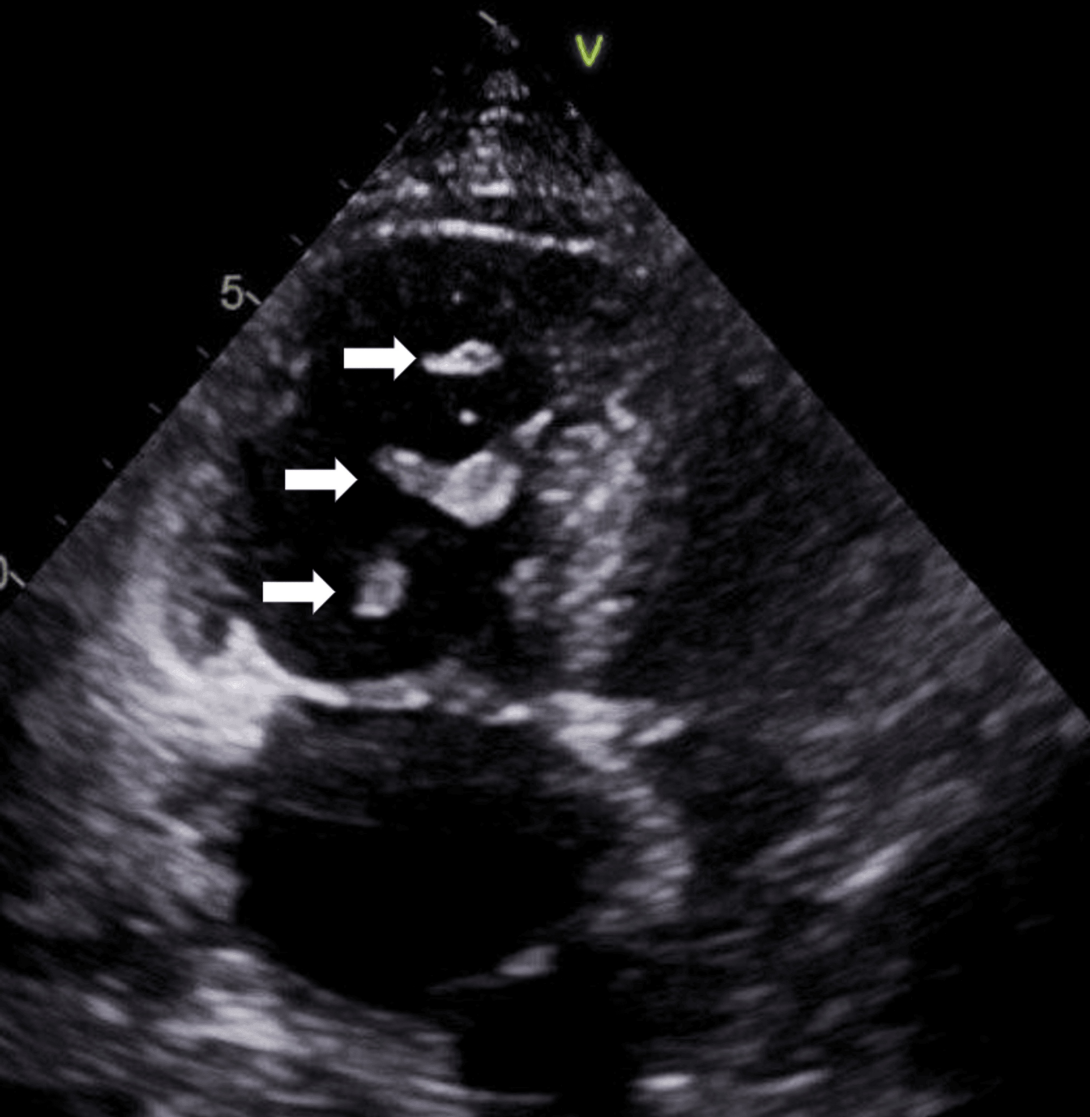
TTE shows an apical four-chamber view (RV focus) showing a thrombus in the RV (white arrows). RV: right ventricular; TTE: transthoracic echocardiogram

**Figure 4:**
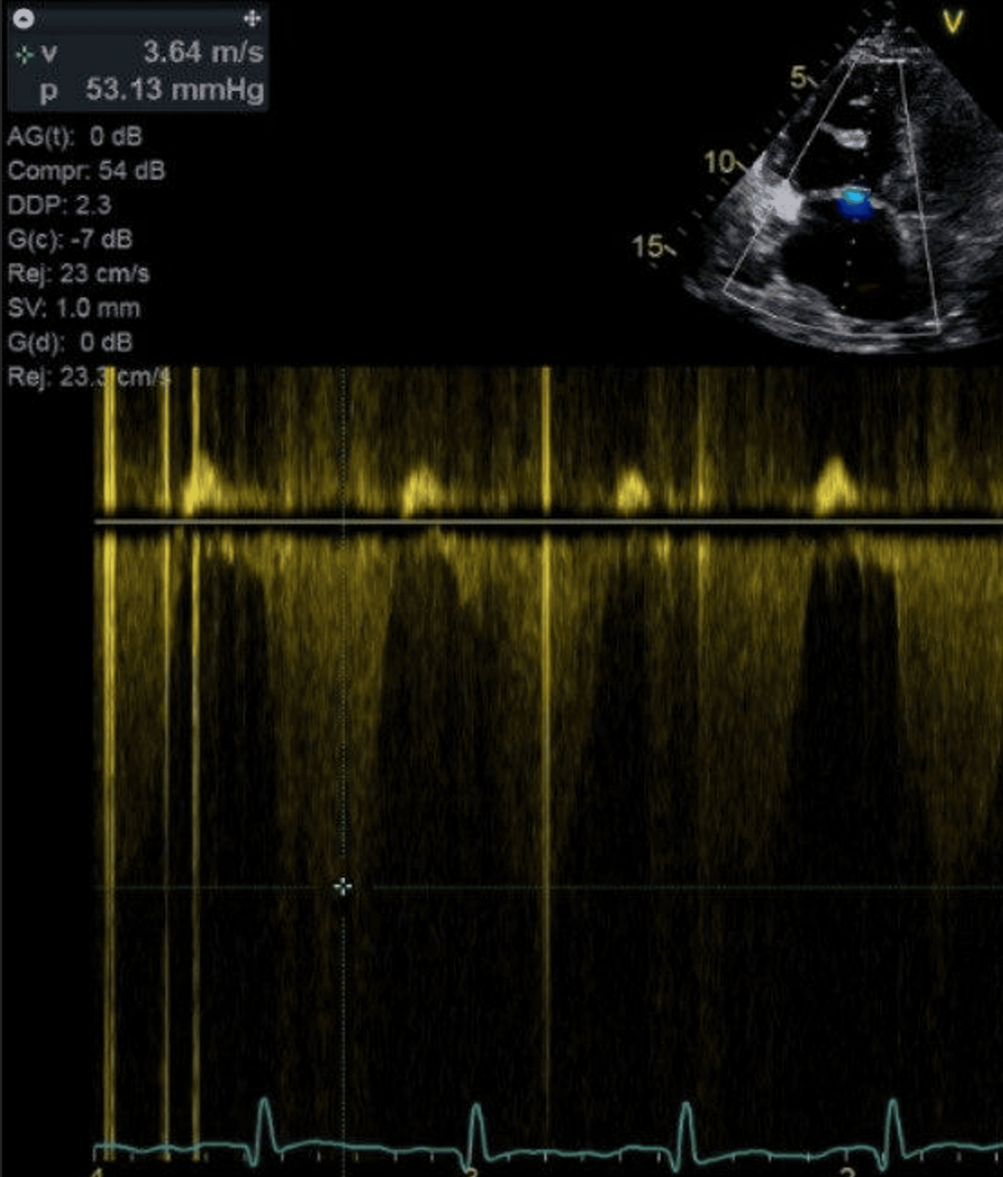
Doppler across the tricuspid valve showing TR with velocity of 3.6 m/sec, indicating an elevated pulmonary pressure. TR: tricuspid regurgitation

TTE showed a preserved left ventricular ejection fraction of 60-65%. The right ventricle was severely dilated with markedly reduced systolic function. A large, mobile echodensity was visualized within the right ventricle, consistent with a clot‑in‑transit (Figure 3). There was akinesis of the right ventricular free wall with apical sparing, consistent with McConnell's sign. The right atrium was severely dilated with leftward bowing of the interatrial septum, suggesting elevated right atrial pressure. Moderate-to-severe tricuspid regurgitation (TR) was present, with an estimated right ventricular systolic pressure of 70 mmHg. The inferior vena cava was dilated with less than 50% inspiratory collapse, consistent with an estimated right atrial pressure of 15 mmHg. Doppler US across the tricuspid valve shows elevated transvalvular pressure, as seen in Figure 4.

Ultrasound Duplex Venous Bilateral Lower Extremities

Ultrasound demonstrated acute deep venous thrombosis involving the external iliac and proximal common femoral veins. Extensive occlusive superficial venous thrombosis was also identified at the saphenofemoral junction and throughout the great saphenous vein from the proximal thigh to the calf.

Management

The pulmonary embolism response team (PERT) was activated. Given her hemodynamic instability and evidence of massive PE with clot‑in‑transit, she underwent emergent percutaneous suction thrombectomy with aspiration of thrombus fragments. Despite aggressive resuscitative efforts, the patient developed worsening obstructive shock and died in the perioperative period.

Case 2

A 74-year-old bed-bound woman with morbid obesity (BMI of 55), atrial fibrillation on rivaroxaban, prior PE, right atrial thrombus status post-surgical thrombectomy (2023), dementia, chronic right lower extremity deep vein thrombosis (DVT), and chronic obstructive pulmonary disease (COPD) on home oxygen presented with acute hypoxemic respiratory failure and altered mental status.

Presentation

**Figure 5:**
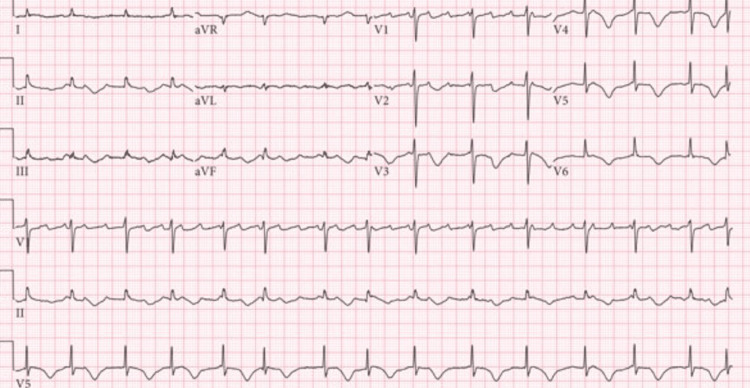
Atrial flutter at a heart rate of around 220 with variable AVN blocks, a ventricular rate of around 90, and inverted T waves (V2-V6). AVN: atrioventricular

**Figure 6:**
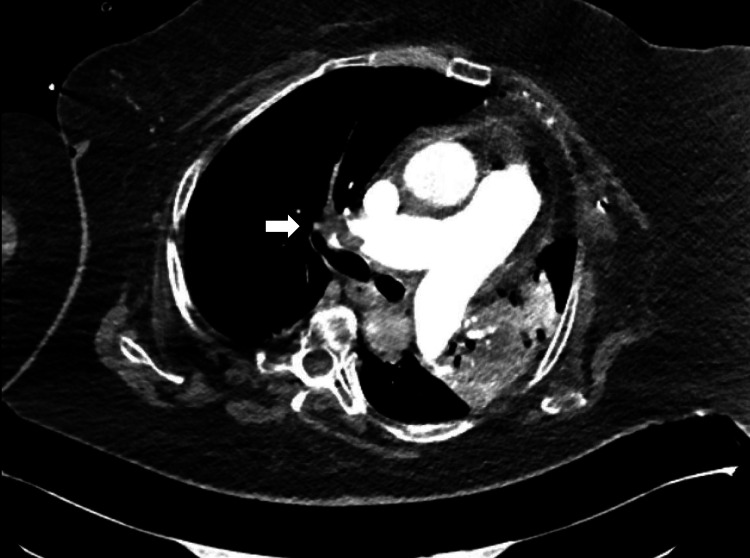
CTPA demonstrated a large right-sided pulmonary embolus (white arrow). CTPA: computed tomography pulmonary angiogram

**Figure 7:**
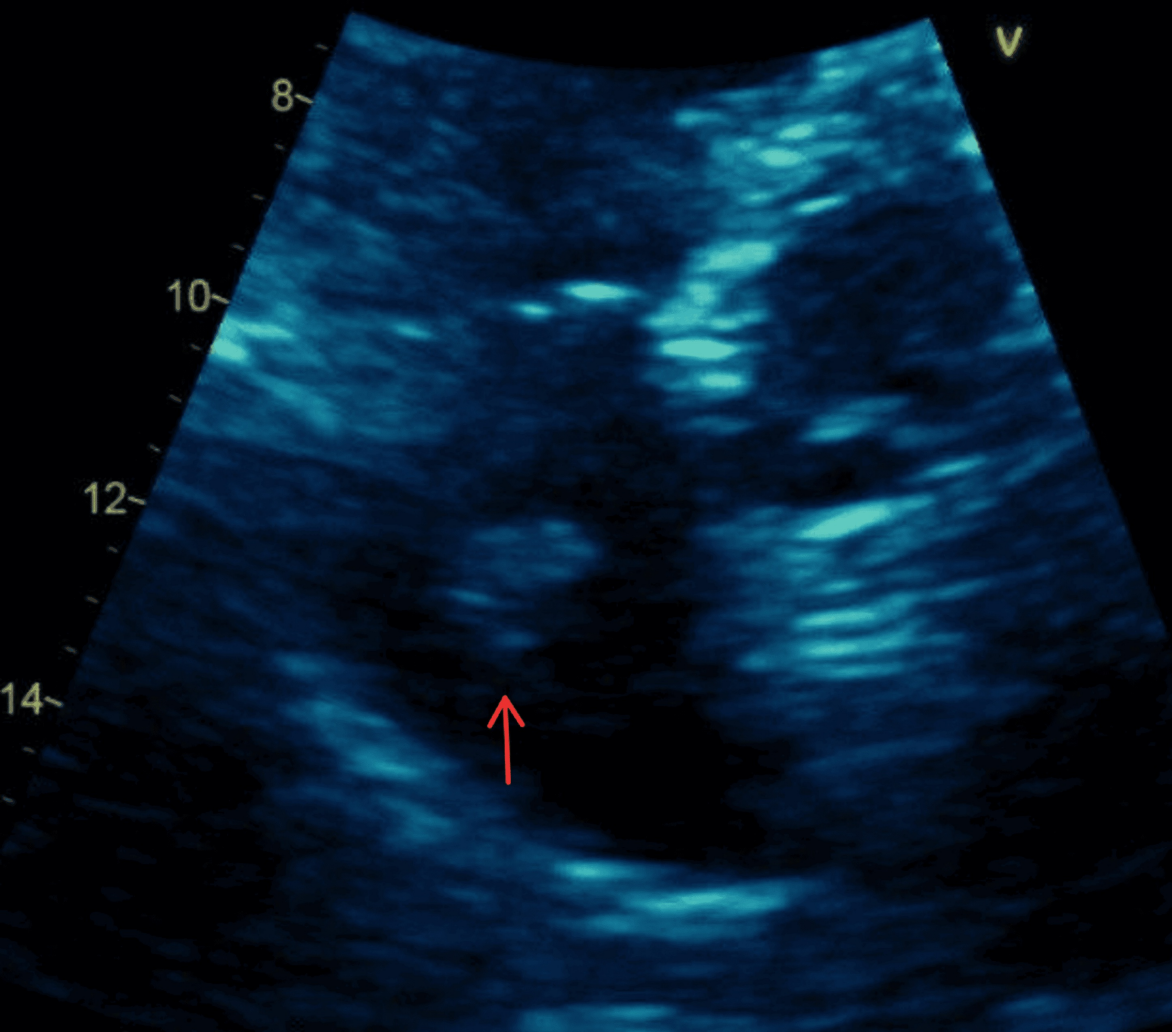
TTE showing the right atrium view with a thrombus in transient (red arrow). TTE: transthoracic echocardiogram

**Figure 8:**
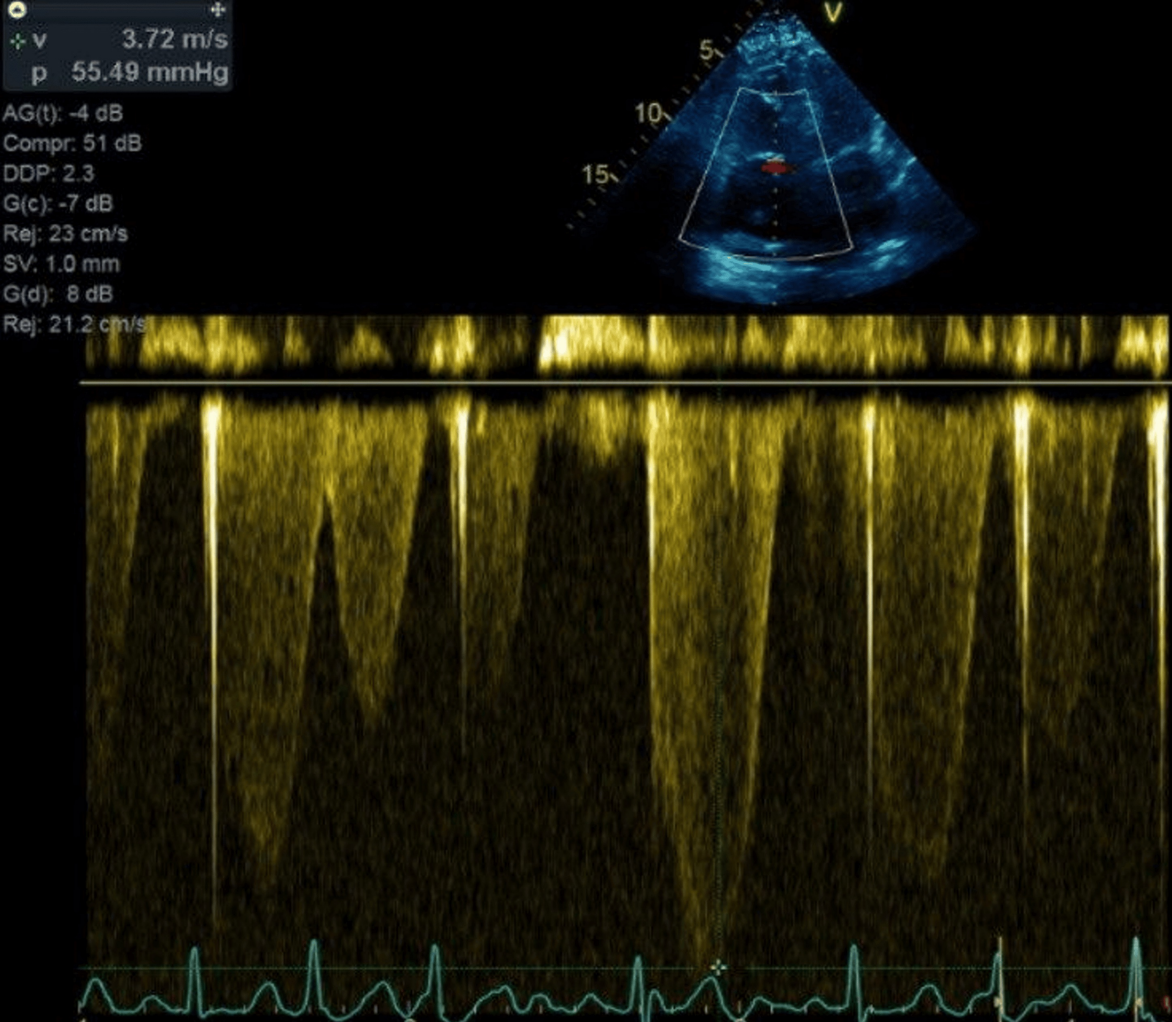
Doppler across the tricuspid valve showing TR with velocity of 3.7 m/sec, indicating an elevated pulmonary pressure. TR: tricuspid regurgitation

She was found at home with severe hypoxemia (SpO_2_ of 70%) and tachycardia (heart rate of 170 bpm). In the emergency department, she was tachypneic with accessory muscle use and was initially placed on bilevel positive airway pressure (BiPAP) but subsequently required endotracheal intubation for airway protection. Chest radiography demonstrated complete opacification of the left hemithorax, concerning for a large pleural effusion. A chest tube was inserted, draining 900 mL of serous fluid. Laboratory findings were significant for leukocytosis, lactic acidosis, and mild hyperkalemia, as presented in Table 2. The EKG was remarkable only for atrial flutter (Figure 5). CT pulmonary angiography was done and was significant for a large right-sided pulmonary embolus and a smaller embolus in the left pulmonary artery, with radiographic evidence of right heart strain (Figure 6). The patient underwent a transesophageal echocardiogram that revealed a large floating thrombus within the right atrium (Figure 7). Similar to the previously discussed case, tricuspid transvalvular Doppler showed elevated pressure across the valve, as seen in Figure 8.

**Table 2:** Laboratory results at admission.

Lab test	Result	Reference range
WBC	18.6 x 10^9^/L	4.5-11.0 x 10^9^/L
Lactate	7.4 mmol/L	0.5-2.2 mmol/L
Potassium	5.4 mmol/L	3.5-5.1 mmol/L

Management and Outcome

The patient was treated with broad-spectrum antibiotics, vasopressor support with norepinephrine, and anticoagulation with argatroban due to a history of suspected heparin-induced thrombocytopenia. Despite maximal medical therapy, she developed progressive shock and ultimately suffered a pulseless electrical activity arrest in the ICU. In accordance with family wishes, resuscitation was not attempted, and she was pronounced deceased later that evening.

## Discussion

TIT represents a medical emergency associated with high early mortality. In both cases, TIT was suspected or confirmed in the setting of acute PE and hemodynamic compromise, highlighting several important considerations.

Epidemiology, mechanism, and prognosis

TIT is identified in approximately 4-18% of acute PE presentations and is consistently associated with high early mortality, particularly among hemodynamically unstable patients [1]. The poor prognosis reflects the underlying pathophysiology: acute PE produces abrupt right ventricular (RV) pressure overload, which impairs RV systolic function and causes interventricular septal shift. This ventricular interdependence reduces left ventricular (LV) filling, leading to systemic hypotension, diminished coronary perfusion, and a downward spiral of RV ischemia and failure. These mechanisms are amplified in the presence of a mobile intracardiac thrombus, which signals a high clot burden and imminent risk of massive embolization.

Prognosis is influenced by clot burden, right ventricular dysfunction, and comorbidities. These cases demonstrate the poor outcome despite taking aggressive measures.

Diagnostic modalities

Echocardiography is the diagnostic modality of choice for detecting TIT, particularly in unstable patients who cannot tolerate transport for advanced imaging. TITs can embolize within seconds and become fatal, so both diagnostics and management must be emergent and involve quick decision-making [4]. Both transthoracic and transesophageal echocardiography can rapidly confirm diagnosis and guide urgent management decisions [3]. CTA remains valuable for diagnosing concomitant PE, as demonstrated in our cases. However, without clear recommendations and guidelines on how to manage TIT, Puello et al. [5] posited that “we must rely on our clinical gestalt” for early detection [5].

Management strategies

Anticoagulation and Systemic Thrombolysis

Anticoagulation alone may be reasonable in hemodynamically stable patients, but carries the risk of embolization and is often inadequate in unstable patients [6]. It has been reported that anticoagulation alone carries the highest mortality compared to other invasive procedures [7]. Systemic thrombolysis offers rapid clot dissolution, but carries a significant bleeding risk, particularly in elderly patients or those with contraindications [4]. Anticoagulation and systemic thrombolysis are considered primary treatment options, while surgical thrombectomy and percutaneous thrombectomy remain as important alternatives in more complicated cases [8].

Surgical Embolectomy

Surgical embolectomy provides definitive clot removal, but perioperative mortality remains high, particularly in unstable patients or those with significant comorbidities [9]. If there is concern regarding immediate thrombus dislodgement, emergent surgical thrombectomies are typically favored over non-surgical therapies [10]. Moreover, patients who come with an extensive clot burden can benefit from surgical thrombectomy, especially if subsequently treated with anticoagulation [11]. However, even in complex scenarios, advanced interventional therapies can be considered too risky. Daly et al. presented a case where treatment with heparin was initiated due to the high-risk nature of invasive procedures [12]; days later, the patient developed new cerebellar infarcts considered to be cardioembolic, alongside hemorrhagic transformation of the left middle cerebral artery that was present on admission, highlighting the delicate balancing act faced by clinicians in these acute scenarios [12].

Percutaneous Thrombectomy

Percutaneous thrombectomy emerges as a less invasive alternative, but its procedural success is variable, and complications include incomplete extraction and embolization [13]. A recent retrospective study found that percutaneous thrombectomy was associated with a significantly lower rate of death and adverse clinical outcomes compared to anticoagulation alone [14].

Overall, available evidence suggests that anticoagulation alone carries the poorest outcomes, while systemic thrombolysis, surgical embolectomy, and percutaneous thrombectomy each offer potential survival benefits in selected patients, emphasizing the need for individualized, risk‑stratified management.

In our series, one patient underwent percutaneous suction thrombectomy but succumbed perioperatively, while the other, managed with anticoagulation and supportive therapy, died from progressive shock. These outcomes reflect the limitations of current therapies in high-risk populations.

Recurrent events and secondary prevention

The second case highlights the challenge of recurrent thromboembolic disease despite chronic anticoagulation. Contributing factors likely included morbid obesity, immobility, and suspected heparin-induced thrombocytopenia, limiting therapeutic options. This underscores the importance of individualized anticoagulant strategies, the role of close follow-up in high-risk patients, and the importance of developing a treatment and management algorithm for TIT.

## Conclusions

TIT is a rare but devastating complication, and it is associated with high mortality despite intervention. We present a side‑by‑side comparison of two patients with nearly identical pathology but different therapeutic approaches, both culminating in poor outcomes. This contrast provides a practical lens into the limitations of current treatment modalities and highlights the importance of rapid diagnosis, multidisciplinary input, and individualized decision‑making. Early recognition with echocardiography, rapid multidisciplinary evaluation, and individualized therapy are essential, but prognosis remains unfavorable. Further prospective research and consensus guidelines are needed to clarify optimal management strategies for this high-risk entity.
